# Multifocal HPV16-positive squamous cell carcinomas in CTLA-4 haploinsufficiency: expanding the spectrum of virus-associated malignancies

**DOI:** 10.1007/s12026-026-09830-x

**Published:** 2026-08-25

**Authors:** Christian Arvei Moen, Kristin Greve-Isdahl Mohn, Alexander Marks, Thea E. Hetland Falkenthal, Patrick Juliebø-Jones, Hans Jørgen Aarstad, Svein Erik Moe, Christian Beisland, Daniela Elena Costea, Elisabet Ognedal, Eirik Bratland, Hildegunn Høberg Vetti, Ellen Berget

**Affiliations:** 1 Department of Urology, Haukeland University Hospital, 5021 Bergen, Norway; 2 Department of Clinical Medicine, University of Bergen, Bergen, Norway; 3 Department of Clinical Medicine, Influenza Centre, University of Bergen, Bergen, Norway; 4 Department of Medicine, Haukeland University Hospital, Bergen, Norway; 5 Cancer Registry of Norway, Norwegian Institute of Public Health, Oslo, Norway; 6 Department of Otolaryngology, Head and Neck Surgery, Haukeland University Hospital, Bergen, Norway; 7 Department of Pathology, Haukeland University Hospital, Bergen, Norway; 8 Department of Medical Genetics, Haukeland University Hospital, Bergen, Norway; 9 Faculty of Health Studies, VID Specialized University, Bergen, Norway

**Keywords:** *CTLA4* variant, Human papillomavirus, Primary immunodeficiency, Autoimmunity, Next-generation sequencing, Squamous cell carcinoma

## Abstract

Cytotoxic T-lymphocyte associated protein 4 (CTLA-4) haploinsufficiency is an inborn error of immunity characterized by immune dysregulation, autoimmunity, lymphoproliferation, and an increased risk of malignancy. While susceptibility to oncogenic viral infections has been proposed, the relationship between CTLA-4 haploinsufficiency and human papillomavirus (HPV)-driven carcinogenesis remains poorly characterized. We describe a patient with CTLA-4 haploinsufficiency who developed multiple HPV-associated malignancies. Clinical, pathological, virological, and genetic data were reviewed. Germline genetic testing was performed using whole-exome sequencing, and HPV DNA analysis was conducted on available tumor specimens. A man with a history of alopecia totalis and autoimmune thyroiditis developed five distinct malignancies by the age of 50 years, including Epstein–Barr virus (EBV)-associated gastric Hodgkin lymphoma, cutaneous squamous cell carcinoma (SCC), and three independent HPV16-positive SCCs involving the oral cavity, anus, and penis. Germline testing identified CTLA-4 haploinsufficiency caused by a likely pathogenic *CTLA4* variant, c.406C > T (p.Pro136Ser). No additional germline variants associated with hereditary cancer predisposition were detected. HPV DNA analysis demonstrated HPV16 positivity in all three mucosal SCCs. This case expands the spectrum of malignancies associated with CTLA-4 haploinsufficiency and suggests that persistent high-risk HPV infection may represent an underrecognized complication of this disorder. The occurrence of three independent HPV16-positive SCCs raises the possibility that CTLA-4 deficiency predisposes to HPV-driven carcinogenesis and supports further investigation of HPV surveillance and cancer prevention strategies in affected individuals.

**Supplementary Information:**

The online version contains supplementary material available at 10.1007/s12026-026-09830-x.

## Introduction

Human papillomavirus (HPV) infection is associated with around 4 – 5% of all cancer-specific deaths worldwide [1]. Consequently, there is an urgent need for a better understanding of HPV-related cancers and development of improved treatment strategies. It is well established that various forms of immunodeficiency (whether primary, secondary, or acquired) are linked to an increased risk of HPV-related disease. However, the mechanisms underlying the development of HPV-associated cancers in immunocompromised individuals remain poorly understood [2].

Cytotoxic T-lymphocyte associated protein 4 (CTLA-4) is an immune checkpoint protein with an inhibitory effect on the interaction between T-cells and antigen-presenting cells (APCs), leading to the downregulation of immune responses [3]. A heterozygous (likely) pathogenic germline variant in the *CTLA4* gene can lead to CTLA-4 haploinsufficiency, an inborn error of immunity characterized by reduced expression and function of CTLA-4. This disorder is associated with a broad spectrum of clinical manifestations, including immune dysregulation, autoimmunity, lymphoproliferation, recurrent infections, and malignancy [4–7]. More than a 100 (likely) pathogenic *CTLA4* variants have been described in the literature and the penetrance of disease has been estimated to be around 70% [6, 7]. As such, a wide spectrum of disease manifestations has been reported in affected individuals [6].

In the context of malignancies, an increased risk of developing Epstein-Barr virus (EBV)-associated lymphoma and gastric cancer has been reported in a cohort of 131 individuals affected by CTLA-4 haploinsufficiency [7]. Within this cohort, one patient was reported to have premalignant intraepithelial neoplasms of the vulva (VIN II) and cervix (CIN II), raising the question of whether these patients may also be more susceptible to chronic infections with HPV. However, invasive HPV-associated squamous cell carcinoma has not been well characterized in this patient population.

Here, we report a patient with CTLA-4 haploinsufficiency who developed five primary malignancies by the age of 50 years, including EBV-associated gastric Hodgkin lymphoma, cutaneous squamous cell carcinoma (SCC), and three independent HPV16-positive SCCs involving the oral cavity, anus, and penis. Based on the clinical, pathological, virological, and genetic findings, we discuss the potential role of CTLA-4 deficiency in persistent HPV infection and multifocal HPV-associated carcinogenesis.

## Methods

### Clinical data collection and patient consent

Following written informed consent, clinical data were retrospectively collected from the patient's medical records. All investigations and treatments were performed at Haukeland University Hospital, Bergen, Norway.

### Germline genetic analyses

To investigate potential genetic causes of the immunodysregulatory phenotype, peripheral blood samples were analyzed by whole-exome sequencing (WES) with gene panel-based filtration of variants in 304 genes associated with primary immunodeficiency (Genomics England PanelApp, Panel ID: 398, version 2.36).

To assess hereditary cancer predisposition, germline DNA was additionally analyzed using the TruSight Hereditary Cancer Panel (Illumina, San Diego, CA, USA), which evaluates sequence variants and copy number variations in 113 cancer predisposition genes (Supplementary Table 1).

### HPV analysis

HPV status was determined in available tumor specimens of all cancers using the multiplex-fluorescent HPV (f-HPV™) typing kit (Genomed Ltd, London, UK). The assay detects HPV types 6, 11, 16, 18, 31, 33, 35, 39, 45, 51, 52, 56, 58, 59, 66, and 68.

### Somatic tumor profiling

The HPV-associated oral, anal, and penile tumors were analyzed using the Oncomine Precision Assay (Thermo Fisher Scientific, Waltham, MA, USA). This targeted next-generation sequencing assay enables detection of hotspot mutations, copy number variations, and gene fusions across 50 cancer-associated genes (Supplementary Table 2).

## Results

### Clinical presentation

The patient had a normal childhood and healthy parents and siblings. There was no known family history of inborn errors of immunity or malignancy. Apart from mild atopic dermatitis, there were no signs of disease until adolescence, when he developed alopecia areata that progressed to alopecia totalis by age 15 years. Immunological evaluation revealed immunoglobulin levels within the lower range of normal. At age 19 years, he was diagnosed with autoimmune thyroiditis.

At age 27 years, he was treated for generalized nummular dermatitis. At the time, it was noted that the patient had genital warts at both the penile and perianal area. During follow-up of his skin condition, it was discovered that the patient had panhypogammaglobulinemia and reduced T- and B-cell counts. Further testing showed that he had undergone infections with Epstein-Barr virus (EBV) and cytomegalovirus (CMV), but not herpes simplex virus (HSV), hepatitis B or C nor human immunodeficiency virus (HIV). A first suspicion of common variable immunodeficiency (CVID) was raised. However, the patient had never suffered from recurrent infections. Empirical treatment with intravenous Ig (Octagam®) was administered. Except for mild hair growth, the patient did not report any effect of this treatment, which was therefore discontinued.

At age 38, he developed a refractory peptic ulcer. Endoscopic biopsies and further investigations revealed stage IVB EBV-positive, HPV-negative gastric Hodgkin lymphoma of mixed cellularity. He was successfully treated with chemoradiotherapy and achieved complete remission.

**Fig. 1:**
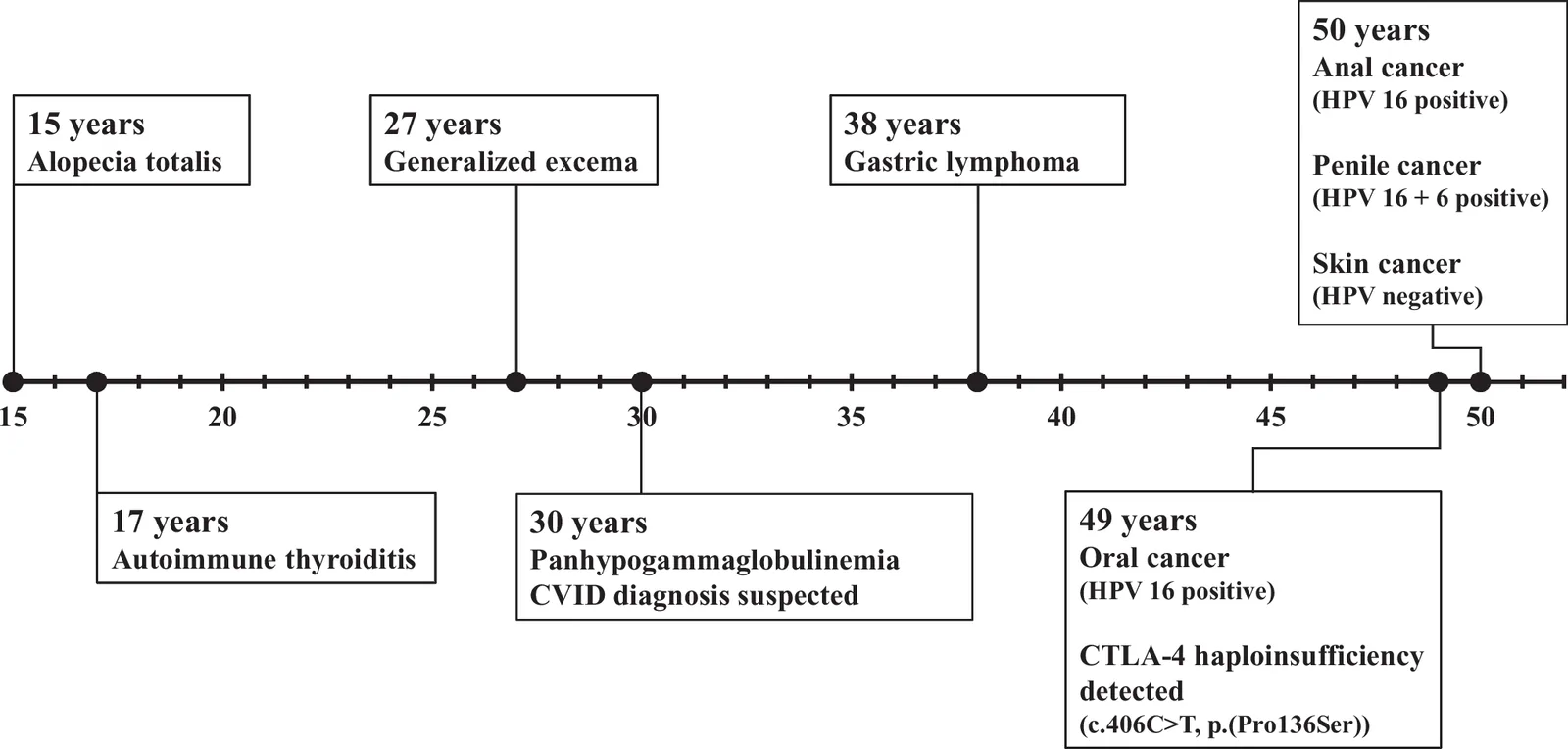
Timeline showing major clinical events. CTLA-4 = Cytotoxic T-lymphocyte associated protein 4, CVID = Common variable immunodeficiency, HPV = Human papillomavirus

**Fig. 2:**
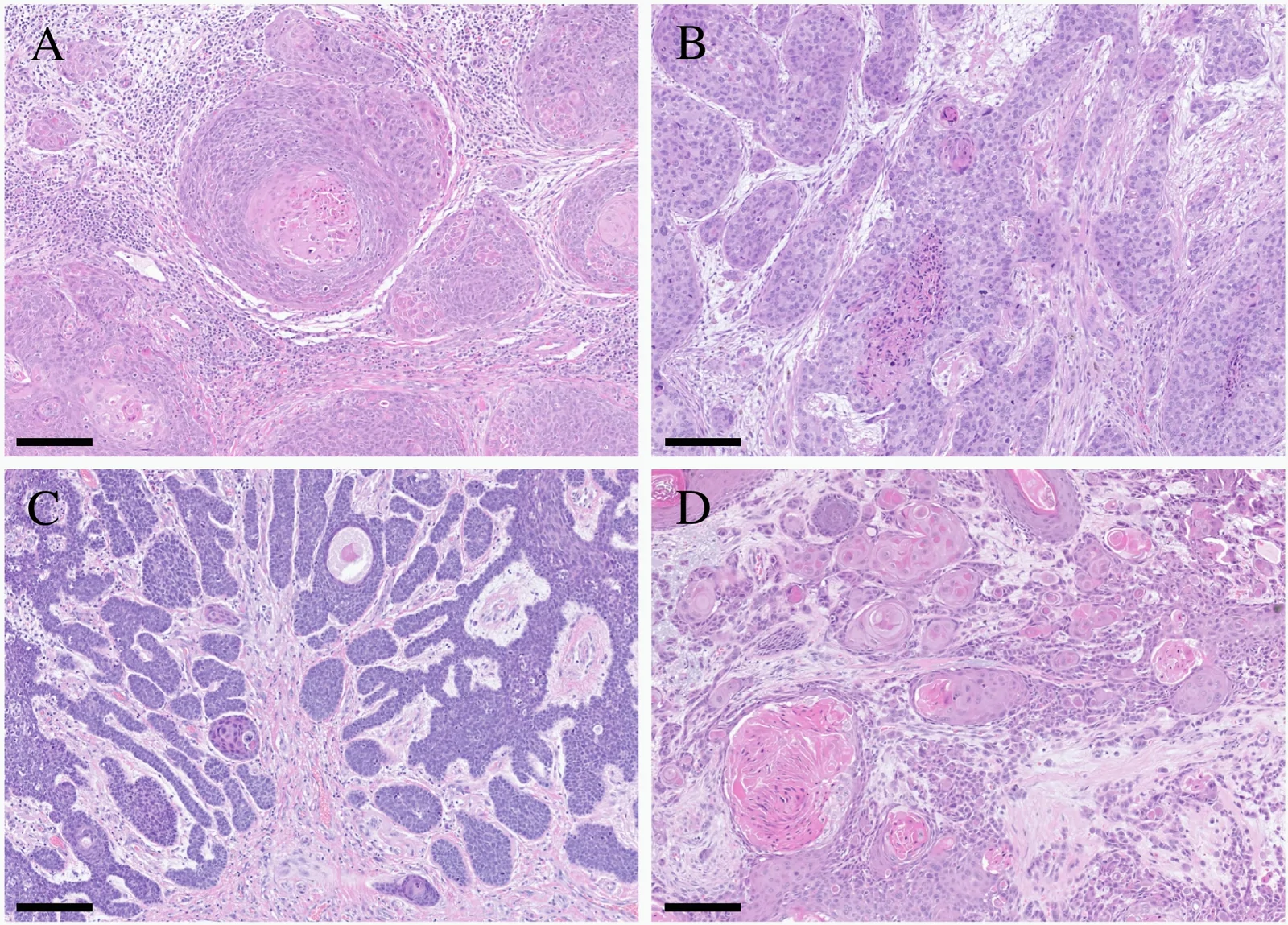
Histopathological images at 40 × magnification. Images showing hematoxylin and eosin (H & E) staining of **A** Oral cancer, **B** Anal cancer, **C** Penile cancer (note the basaloid features) and **D** Skin cancer. A scale bar of 200 µm is shown in each panel

At age 48 he sought medical advice for his perianal genital warts. The patient was referred to a specialist, and he also received three doses of vaccination against HPV infection (Gardasil®9). Concomitantly, however, a biopsy from an ulcer in the gingiva of the inferior oral cavity revealed an HPV 16 positive SCC. He underwent surgery for his oral cancer (pT3N0), followed by adjuvant radiation therapy. Following recovery of his oral cancer, surgery for his perianal lesions revealed an HPV 16 positive anal SCC (pT3Nx), for which he underwent adjuvant chemoradiotherapy. He was then referred to urological examination of his penile genital warts, which again revealed HPV 16 and HPV 6 positive penile SCC (pT1aN0). His penile cancer was successfully treated with surgery alone. While undergoing treatment for penile cancer, a biopsy of an ulcer on the cheek revealed an HPV negative skin SCC (with histopathological indirect signs of sun-damaged dermis) which additionally was surgically excised with free resection margins (pT1Nx). A summary of the patient's clinical course and major disease events is shown in Fig. 1. Histopathological images of the four SCCs are shown in Fig. 2.

### Germline genetic findings

While undergoing treatment for his oral cancer, repeat immunological investigations confirmed panhypogammaglobulinemia and low levels of both T- and B-cells. WES analysis of possible primary immunological disorders revealed a likely pathogenic heterozygous missense variant CTLA4 (NM_005214.5): c.406C > T resulting in an amino acid substitution p. (Pro136Ser). This variant lies within the highly conserved MYPPPY motif of the *CTLA4* gene, constituting a possible hotspot for pathogenic missense variants. No additional germline variants associated with hereditary cancer predisposition were identified using the TruSight Hereditary Cancer Panel.

### HPV status and tumor genomic profiling

HPV DNA analysis demonstrated HPV16 positivity in the oral, anal, and penile squamous cell carcinomas. The penile carcinoma was additionally positive for HPV6. The gastric Hodgkin lymphoma and cutaneous squamous cell carcinoma were HPV DNA negative.

Targeted genomic profiling of the HPV-associated tumors did not identify pathogenic somatic alterations within the genes included in the Oncomine Precision Assay.

### Follow-up

Following completion of cancer treatment, the patient received empirical treatment with the CTLA-4–Ig fusion protein abatacept for six months without clear clinical benefit. The patient also received counseling regarding hematopoietic stem cell transplantation (HSCT) as a potential treatment option for CTLA-4 haploinsufficiency, but did not wish to pursue this further. At the latest follow-up, he remained recurrence-free after two years of oncological surveillance.

## Discussion

CTLA-4 haploinsufficiency is associated with immune dysregulation and an increased risk of malignancy, particularly EBV-associated lymphoma and gastric cancer. However, the relationship between CTLA-4 deficiency and HPV-driven carcinogenesis remains poorly characterized. In the present case, a patient with CTLA-4 haploinsufficiency developed three independent HPV16-positive squamous cell carcinomas involving the oral cavity, anus, and penis. Given the rarity of multiple HPV-associated cancers, these findings support the hypothesis that impaired immune control of HPV may represent an underrecognized manifestation of CTLA-4 haploinsufficiency.

Common *CTLA4* genetic polymorphisms have previously been associated with variable susceptibility to HPV 16-induced cervical cancer [8]. This suggests that not only the availability, but also the regulation of CTLA-4 protein expression may influence the risk of developing HPV-related cancer. The current case harbored a likely pathogenic missense variant *CTLA4* c.406C > T p.(Pro136Ser), located adjacent to the (likely) pathogenic *CTLA4* variant c.410C > T p.(Pro137Leu) reported in a woman with both CIN and VIN [7]. These variants are both located within the region in exon 2 of the *CTLA4* gene that encodes the highly conserved MYPPPY motif, an area of the extracellular domain of CTLA-4 that is critical for binding to CD80/86 on APCs [3, 6, 9–11]. Although functional studies were not performed, disruption of this interaction could impair CTLA-4-mediated immune regulation and contribute to defective immune control of persistent HPV infection.

Once exposed to HPV, it is well established that the HPV virus itself may employ multiple immune evasion strategies to avoid host immune responses [12]. Of note, HPV oncogenes are known to modulate the interaction between CTLA-4 on T-cells and CD80/86 on APCs [13]. Assuming that this interaction is already disturbed in patients with CTLA-4 haploinsufficiency, it may further help explain their potentially increased susceptibility to persistent HPV infection. Moreover, it has also been suggested that HPV may induce expression of CTLA-4 in infected keratinocytes, which may subsequently inhibit nearby APCs or result in an immunosuppressive environment surrounding the infected cells [14]. Together, multiple factors directly related to CTLA-4 may help explain why this specific patient developed persistent HPV infections at three independent sites.

There were no somatic mutations identified in either of the HPV related tumor tissues, and the patient had no additional germline variants associated with hereditary risk of cancer development. This supports the notion that these HPV-related cancers were primarily driven by viral oncogenes. However, in this setting, more comprehensive analyses of the tumor tissue, such as whole-genome or whole-exome sequencing, could have provided additional information.

The cutaneous SCC was negative for all α-papillomavirus types included in the HPV assay. However, β-papillomaviruses are more commonly associated with cutaneous epithelial surfaces and have been implicated in the development of cutaneous SCC [15–17]. Because β-papillomavirus types were not assessed in the present study, a potential contribution of HPV to the skin cancer cannot be excluded, although such a role remains speculative. However, if this was indeed the case, then all his five cancers can be associated with viral infections.

Multiple HPV associated cancers are reported to be exceedingly rare [18–20]. The occurrence of three HPV16-positive SCCs affecting distinct anatomical sites in a single patient strongly suggests an underlying defect in immune control of HPV infection. Together with previous reports of HPV-associated premalignant lesions in CTLA-4 haploinsufficiency, the present case supports the hypothesis that persistent HPV infection may represent a clinically relevant manifestation of this disorder.

One issue complicating the research in CTLA-4 haploinsufficiency is the large variety of phenotypic presentations, even among individuals with the exact same variant [6, 7]. This variability suggests that additional genetic, epigenetic, environmental, and viral factors influence disease expression and needs to be taken into consideration to fully understand the outcomes of this condition [4, 5]. Of note, recent work has suggested that HLA genotype may contribute to the phenotypic heterogeneity observed in CTLA-4 insufficiency. Although no association with overall disease onset or severity was identified, specific HLA alleles were associated with particular clinical manifestations, supporting the concept that host genetic factors may modify disease expression [21]. Possibly, the interplay between these factors could also explain the large variation in the age of onset among patients [6], and also why the patient reported here had normal levels of immunoglobulins in his youth, but low levels later in life. Moreover, future studies are needed to also determine whether susceptibility to persistent HPV infection and HPV-associated malignancies varies according to the underlying *CTLA4* variant.

## Conclusion

This case expands the spectrum of malignancies associated with CTLA-4 haploinsufficiency and provides further evidence that impaired immune control of HPV may contribute to disease manifestations in affected individuals. The occurrence of three independent HPV16-positive squamous cell carcinomas of the oral cavity, anus, and penis suggests that persistent high-risk HPV infection may represent an underrecognized complication of CTLA-4 insufficiency. Further studies are needed to clarify the relationship between CTLA-4 haploinsufficiency and HPV-driven carcinogenesis and to determine whether prophylactic HPV vaccination should be routinely considered and offered to patients with CTLA-4 insufficiency.

## Supplementary Information

Below is the link to the electronic supplementary material.

Supplementary file1 (DOCX 29 KB)

Supplementary file2 (DOCX 16 KB)

## Data Availability

The data supporting the findings of this study are available from the corresponding author upon request. The data are not publicly available due to privacy and ethical restrictions.
