# Supplementary material for: Multifocal HPV16-positive squamous cell carcinomas in CTLA-4 haploinsufficiency: expanding the spectrum of virus-associated malignancies
Source: Immunol Res. 2026 Aug 25;74(1):95. doi: 10.1007/s12026-026-09830-x (PMC13506675; doi:10.1007/s12026-026-09830-x)
Supplement: Supplementary file 1 [file 12026_2026_9830_MOESM1_ESM.docx]

| *ACD* | *DIS3L2* | *GREM1* | *PIK3CA* | *SDHD* |
| --- | --- | --- | --- | --- |
| *AIP* | *EPCAM* | *HOXB13* | *PMS2* | *SLX4* |
| *AKT1* | *ERCC1* | *KIF1B* | *POLD1* | *SMAD4* |
| *APC* | *ERCC2* | *KIT* | *POLE* | *SMARCA4* |
| *ATM* | *ERCC3* | *LZTR1* | *POT1* | *SMARCB1* |
| *BAP1* | *ERCC4* | *MAX* | *PRKAR1A* | *SMARCE1* |
| *BARD1* | *ERCC5* | *MEN1* | *PTCH1* | *SPINK1* |
| *BLM* | *FAM175A* | *MET* | *PTEN* | *SPRED1* |
| *BMPR1A* | *FANCA* | *MITF* | *RAD50* | *STK11* |
| *BRCA1* | *FANCB* | *MLH1* | *RAD51* | *SUFU* |
| *BRCA2* | *FANCC* | *MRE11A* | *RAD51B* | *TERF2IP* |
| *BRIP1* | *FANCD2* | *MSH2* | *RAD51C* | *TERT* |
| *CASR* | *FANCE* | *MSH3* | *RAD51D* | *TMEM127* |
| *CDC73* | *FANCF* | *MSH6* | *RB1* | *TP53* |
| *CDH1* | *FANCG* | *MUTYH* | *RECQL4* | *TSC1* |
| *CDK4* | *FANCI* | *NBN* | *RET* | *TSC2* |
| *CDKN1B* | *FANCL* | *NF1* | *RHBDF2* | *VHL* |
| *CDKN2A* | *FANCM* | *NF2* | *RINT1* | *WT1* |
| *CEBPA* | *FH* | *NSD1* | *RUNX1* | *XPA* |
| *CHEK2* | *FLCN* | *NTHL1* | *SDHA* | *XPC* |
| *CTRC* | *GALNT12* | *PALB2* | *SDHAF2* | *XRCC2* |
| *DDB2* | *GATA2* | *PDGFRA* | *SDHB* |  |
| *DICER1* | *GPC3* | *PHOX2B* | *SDHC* |  |

Supplementary Table 1. TruSight Hereditary Cancer Panel
