# Supplementary material for: Multifocal HPV16-positive squamous cell carcinomas in CTLA-4 haploinsufficiency: expanding the spectrum of virus-associated malignancies
Source: Immunol Res. 2026 Aug 25;74(1):95. doi: 10.1007/s12026-026-09830-x (PMC13506675; doi:10.1007/s12026-026-09830-x)
Supplement: Supplementary file 2 [file 12026_2026_9830_MOESM2_ESM.docx]

| **DNA hotspot mutations** | | | | |
| --- | --- | --- | --- | --- |
| *AKT1* | *CHEK2* | *FGFR3* | *KIT* | *NTRK3* |
| *AKT2* | *CTNNB1* | *FGFR4* | *KRAS* | *PDGFRA* |
| *AKT3* | *EGFR* | *FLT3* | *MAP2K1* | *PIK3CA* |
| *ALK* | *ERBB2* | *GNA11* | *MAP2K2* | *PTEN* |
| *AR* | *ERBB3* | *GNAQ* | *MET* | *RAF1* |
| *ARAF* | *ERBB4* | *GNAS* | *MTOR* | *RET* |
| *BRAF* | *ESR1* | *HRAS* | *NRAS* | *ROS1* |
| *CDK4* | *FGFR1* | *IDH1* | *NTRK1* | *SMO* |
| *CDKN2A* | *FGFR2* | *IDH2* | *NTRK2* | *TP53* |
| **CNVs** | | **Gene fusions** | | |
| *ALK* | *FGFR1* | *ALK* | *FGFR2* | *NTRK3* |
| *AR* | *FGFR2* | *AR* | *FGFR3* | *NUTM1* |
| *CD274* | *FGFR3* | *BRAF* | *MET* | *RET* |
| *CDKN2A* | *KRAS* | *EGFR* | *NRG1* | *ROS1* |
| *EGFR* | *MET* | *ESR1* | *NTRK1* | *RSPO2* |
| *ERBB2* | *PIK3CA* | *FGFR1* | *NTRK2* | *RSPO3* |
| *ERBB3* | *PTEN* |  |  |  |

Supplementary Table 2. Oncomine precision assay gene list

CNVs = Copy number variations
